# Lone-Pair-Enabled Polymorphism and Photostructural Changes in Chalcogenide Glasses

**DOI:** 10.3390/ma16196602

**Published:** 2023-10-09

**Authors:** Alexander V. Kolobov, Vladimir G. Kuznetsov, Milos Krbal, Stanislav V. Zabotnov

**Affiliations:** 1 Institute of Physics, Herzen State Pedagogical University of Russia, 48 Moïka Emb., St. Petersburg 191186, Russia; 2 Ioffe Institute, 26 Polytechnicheskaya Street, St. Petersburg 191186, Russia; vladimir.kuznetsov@mail.ioffe.ru; 3 Center of Materials and Nanotechnologies, Faculty of Chemical Technology, University of Pardubice, Nam. Čs. Legii 565, 530 02 Pardubice, Czech Republic; milos.krbal@upce.cz; 4 Faculty of Physics, Lomonosov Moscow State University, 1/2 Leninskie Gory, Moscow 119991, Russia

**Keywords:** chalcogenide glasses, photostructural changes, polymorphism, density functional theory

## Abstract

S- and Se-based chalcogenide glasses are intrinsically metastable and exhibit a number of photo-induced effects unique to this class of materials, reversible photostructural changes and photo-induced anisotropy being major examples. These effects are usually interpreted in terms of the formation of valence alternation pairs and ‘wrong’ bonds. In this work, using density functional theory simulations, we demonstrate for the case example of ${\mathrm{As}}_{2}S_{3}$ that a strong decrease in the optical band gap can be achieved if a polymorphic transformation of the local structure from orpiment to that of tetradymite takes place. For the formation of the latter, the presence of lone-pair electrons in near-linear atomic configurations is crucial. Our results represent a novel approach to understanding the photo-induced structural changes in chalcogenide glasses as being due to the presence of polymorphism, and will lead to their wider use in various photonic devices.

## 1. Introduction

Tellurium-based chalcogenides, also known as phase-change alloys such as GeTe and ${\mathrm{Sb}}_{2}{\mathrm{Te}}_{3}$, are widely used in optical memory devices (compact discs, digital versatile discs and Blu-ray discs) as well as in so-called phase-change random access memory (PC-RAM) devices. In these applications, the information is stored in the structure of the material, which can be controllably changed during a reversible transition between the amorphous and crystalline states. The amorphous phase (RESET state) is less reflective and highly resistant, while the crystalline phase is more reflective and less resistant. The capability of these chalcogenides to undergo a fast and reversible crystallization–amorphization process is due to the poor glass-forming ability of Te-based chalcogenides.

Chalcogenides, based on lighter elements such as sulfur or selenium, on the other hand, are good glass formers and form very stable glasses. As such, being intrinsically metastable, their structure can be reconfigured within the amorphous phase. Consequently, chalcogenide glasses, of which ${\mathrm{As}}_{2}S_{3}$ is a classic representative, exhibit a number of photo-induced phenomena, such as reversible photostructural change, photo-induced anisotropy, photo-induced volume expansion, etc. (for reviews, see [1,2]). Reversible photostructural change, experimentally detected by structure-sensitive techniques such as Raman scattering [3], X-ray scattering [4] and extended X-ray absorption fine structure (EXAFS) [5,6], manifests itself as photodarkening [7] when the optical band gap decreases upon illumination. The initial properties can be restored by subsequent annealing at temperatures close to the glass-transition temperature. The band gap decrease depends on the material composition and can reach a value as large as 0.3 eV [8], which is close to 15% of the initial gap value. The photodarkening is usually accompanied by volume expansion [9,10]. When glasses are exposed to linearly polarized light, photo-induced dichroism and birefringence are also observed [11]. The photo-induced anisotropy can be reoriented if the polarization of the light is changed [12]. In addition, chalcogenide glasses are highly transparent into the infrared region of light.

These properties enable numerous photonic applications [13,14,15]. The high transparency of chalcogenide glasses in the spectral region of 2–25 μm makes them an attractive material for optical fibers and waveguides for communications and optical systems [16]. The high material non-linearity opens up the possibility of their use in non-linear optical devices [17]. Photodarkening, which is accompanied by a change in refractive index, and allows one to engineer the properties of these materials in a controllable way, can be used to create waveguides in evaporated films [18], fabricate Bragg gratings [19] and distributed laser mirrors [20], to name a few. The photo-induced change in chemical reactivity makes them high-resolution photoresists [21,22]. Surface modification such as photoexpansion [10,23,24] allows one to fabricate lenses without the need to etch the materials. Of special interest is direct surface relief laser writing on chalcogenide glasses such as ${\mathrm{As}}_{2}S_{3}$ using femto-second laser light [25,26,27,28].

In order to develop novel applications of photo-induced phenomena in chalcogenide glasses, an atomistic-level understanding of structural changes is needed. Various models have been proposed to account for the observed changes, such as bond switching [29], the formation of photo-induced valence alternation pairs [5] and the formation of ‘wrong’ bonds [3]. It was also proposed that so-called soft modes may serve to generate states with negative correlation energy responsible for photodarkening [30]. Computer studies also concluded that interchain interaction was enhanced in selenium as evidenced by the formation of dynamic covalent bonds between neighboring layers [31]. In this work, we propose a novel approach to photostructural changes based on a lone-pair-enabled polymorphism of the local structure.

## 2. Simulation Details

Density functional calculations of the orpiment and tetradymite phases were carried out at 0 K using the plane-wave pseudopotential-based code CASTEP [32]. The Vanderbilt ultrasoft scalar relativistic pseudopotentials [33] were chosen to describe electron–ion interactions. The As 4s^2^4p^3^ and S 3s^2^3p^4^ electrons were assigned to valence electrons. The exchange-correlation term was evaluated within the generalized gradient approximation (GGA) using the PBE functional [34] as implemented in CASTEP. The cut-off energy of 330 eV was used, based on a convergence study. The simultaneous convergence criteria were set to the following values: energy—5 × 10^−6^ eV/atom; max. force—0.01 eV/A; max. stress—0.02 GPa; max. displacement—5 × 10^−4^ Å. Phonon dispersion spectra were calculated for equilibrium geometry using the linear response approach or density functional perturbation theory (DFPT) [35] and its CASTEP implementation within the plane-wave pseudopotential formalism [36].

The amorphous phase was generated by the following ‘melt-quench’ procedure [37]. The initial (crystalline) structure was randomized by heating the structure to 3000 K over a period of 20 ps followed by cooling to a temperature just above the melting point 900 K over a period of 45 ps followed by quenching from 900 to 300 K over a duration of 15 ps. essentially A total of 240 atoms were included in the simulation cell with a density 3.49 g/cm^3^. Ab initio molecular dynamics calculations were carried out using the plane-wave pseudopotential code VASP using an NVT ensemble [38] with a Nosé thermostat. The gamma point and a Gaussian smearing of 0.05 eV were used for Brillouin zone integration. A value of 260 eV was chosen for the plane-wave cutoff as recommended by the VASP developers. The Perdew–Burke–Ernzerhof exchange-correlation functional was utilized [34]. The projected-augmented wave method [39], in which the outermost *s*-and *p*-electrons were treated as valence electrons, was used to correct for the effect of the core electrons within the augmentation sphere [40].

## 3. Results and Discussion

First of all, we ask the question ‘Why the absorption changes under photoexcitation in some glassy materials but not in others?’ The answer, we believe, is linked to the nature of the band gap in glasses. The gap in crystals, in most treatments, is closely related to the Bragg reflection of the electron waves by the crystal lattice (at edges of the Brillouin zone) and its mathematical treatment is based on the assumption of long-range order in a perfect crystal. Glasses do not have a periodic structure and consequently do not give a sharp Bragg reflection. Then, “how can glass be transparent?” asks Sir Nevill Mott in his Nobel lecture [41]. An answer to this question can be traced to A.F. Ioffe [42], who proposed that optical properties of materials are primarily determined by the short-range order. It is not surprising, therefore, that most glasses possess properties very similar to their crystalline counterparts. This fact has long been known as the Zachariasen paradigm [43], stating that, if a glass is formed from a crystal, their optical properties are fairly the same, since glasses must be considered as the media maintaining the local bonding structure of their parent crystals and losing only the long-range order.

The crucial point of the Zachariasen paradigm is that the glass “maintains the local bonding structure of the parent crystal”. But what happens if a glass is a ‘child’ of several different ‘parent’ crystals? Obviously, such a glass may have fragments with local structures reminiscent of different crystalline phases, or different polymorphs. In this case, the properties of the glass will be determined by the crystalline polymorph whose local structure the glassy phase inherited. Since electronic excitation can cause transitions between different crystalline polymorphs [44,45], it is not unnatural to assume that a similar process can take place in a glassy phase. In other words, we argue that a precondition for photostructural changes to occur in glasses is the presence of polymorphism in the related crystals.

**Figure 1:**
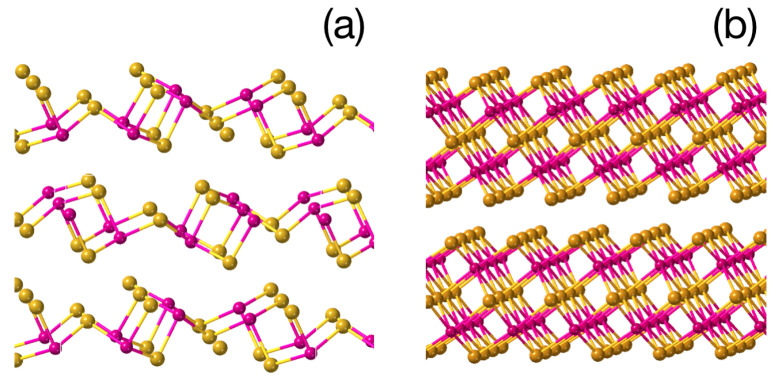
The orpiment (**a**) and tetradymite (**b**) structures of $A_{2}^{V}B_{3}^{\mathrm{VI}}$. Pnictogen atoms are shown in violet and chalcogen atoms are yellow.

In this section, we consider the effect of the bonding nature and local bonding geometry in ${\mathrm{As}}_{2}S_{3}$ on the phase stability and optical properties of the material. Because, as mentioned above, the local structure of an amorphous material usually resembles that of the corresponding crystal [43,46], we model the local structure of the glass by those of the corresponding crystalline polymorphs. Here, it is interesting to note that while crystals such as ${\mathrm{As}}_{2}S_{3}$ and ${\mathrm{As}}_{2}{\mathrm{Se}}_{3}$ possess the orpiment structure, their isoelectronic homologues ${\mathrm{Sb}}_{2}{\mathrm{Te}}_{3}$, ${\mathrm{Bi}}_{2}{\mathrm{Se}}_{3}$ and ${\mathrm{Bi}}_{2}{\mathrm{Te}}_{3}$ crystallize into a layered tetradymite structure. The latter attracted much attention for being robust three-dimensional topological insulators [47]. In Figure 1, we show the orpiment (left) and tetradymite (right) structure of $A_{2}^{V}B_{3}^{\mathrm{VI}}$, where yellow circles correspond to chalcogen atoms and violet circles depict pnictogens.

As mentioned above, these two groups of $A_{2}^{V}B_{3}^{\mathrm{VI}}$ materials are isoelectronic but form very different structures. In the orpiment structure, covalently bonded fragments are formed with all elements satisfying the 8–N rule [48], i.e., chalcogen atoms are two-fold coordinated and pnictogens are three-fold coordinated. The interaction between covalently bonded blocks is usually described as being of van-der-Waals type. Heavier chalcogenides and pnictides typically form a tetradymite structure based on quintuple layers (QLs) with the stacking sequence of C-P-C-P-C, where C stands for chalcogens and P corresponds to pnictogens. The outermost chalcogen atoms in the quintuple layer are three-fold coordinated, with all other atoms being octahedrally coordinated. In both cases, the 8–N rule is violated.

The octahedral local coordination in the tetradymite structure is enabled by the presence of *p*-orbitals that can serve to form covalent bonds. For example, in $A^{\mathrm{IV}}B^{\mathrm{VI}}$ crystals, the cubic (rhombohedrally distorted, to be exact) structure can be formed if Te atoms formally donate two electrons to a group IV element, such as Ge or Sn. The combination of a Te lone-pair and an empty *p*-orbital of Ge (Sn) allows for the formation of a dative bond, making both Te and Ge (Sn) three-fold coordinated. The details can be found in [49,50]. Interaction between back-lobes of the *p*-orbitals involved in covalent bonding leads to the creation of resonant (or metavalent) bonds [51,52], making participating atoms six-fold coordinated. Similar multicenter bonds formed due to the presence of lone-pair electrons and back-lobes of p-orbitals are responsible for the formation of quintuple layers in materials such as ${\mathrm{Sb}}_{2}{\mathrm{Te}}_{3}$.

**Figure 2:**
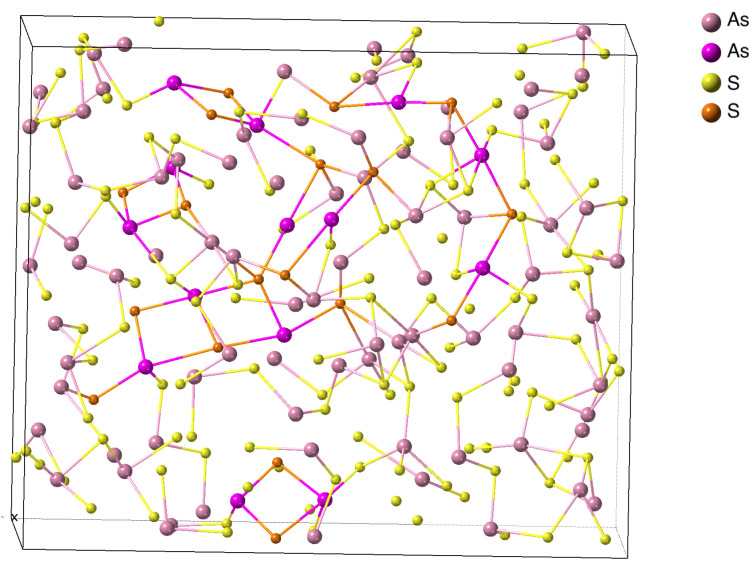
In silico melt-quenched amorphous ${\mathrm{As}}_{2}S_{3}$. In addition to fragments satisfying the 8–N rule, linear fragments marked by brighter colours and As-S-As-S squares are clearly visible.

In Figure 2, we show an in silico amorphous ${\mathrm{As}}_{2}S_{3}$ structure obtained using the procedure described above. One can see that, indeed, in addition to ${\mathrm{AsS}}_{3}$ units characteristic of the crystalline orpiment phase, linear atomic fragments (in some cases as long as five atoms) and As-S-As-S square rings reminiscent of the tetradymite phase are formed. (The tetradymite fragments are shown in somewhat brighter colours in the figure). This result demonstrates the suitability of the proposed concept. Interestingly, such extended linear fragments were also found in the amorphous phase of ${\mathrm{Sb}}_{2}{\mathrm{Te}}_{3}$ [53]. It should also be noted that similar extended fragments, called hypervalent bonds, formed due to the presence of lone-pair electrons, were introduced by Dembovsky and Chechetkina [54] as major defects in chalcogenide glasses.

**Figure 3:**
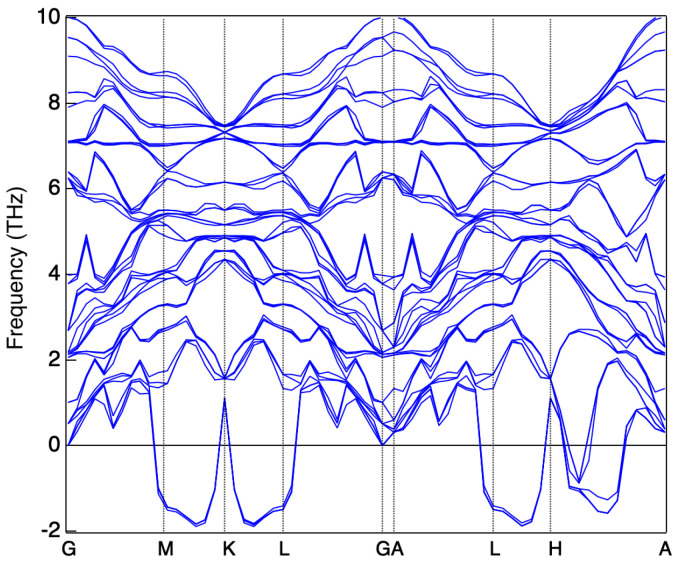
Phonon dispersion spectra calculated for the tetradymite phase of ${\mathrm{As}}_{2}S_{3}$. The presence of imaginary modes (shown as negative in the plot) is an indication of the phase instability.

We now proceed to the stability and properties of different ${\mathrm{As}}_{2}S_{3}$ structures. The orpiment and tetradymite structures of ${\mathrm{As}}_{2}S_{3}$ optimized at 0 K are shown in Figure 1 and possess an energy difference of 0.7 eV per ${\mathrm{As}}_{2}S_{3}$ formula unit with the stable phase characterized by the orpiment structure. The rather large energy difference ensures that, in the ground state of the glass, the fraction of QL-like building units is negligibly small. The bulk tetradymite phase of ${\mathrm{As}}_{2}S_{3}$, on the other hand, is characterized by a phonon dispersion spectrum that contains imaginary modes (Figure 3), i.e., it is not stable. We suppose, however, that fragments of this structure may be created in the photo-excited state and survive surrounded by the orpiment-like network of the glass.

**Figure 4:**
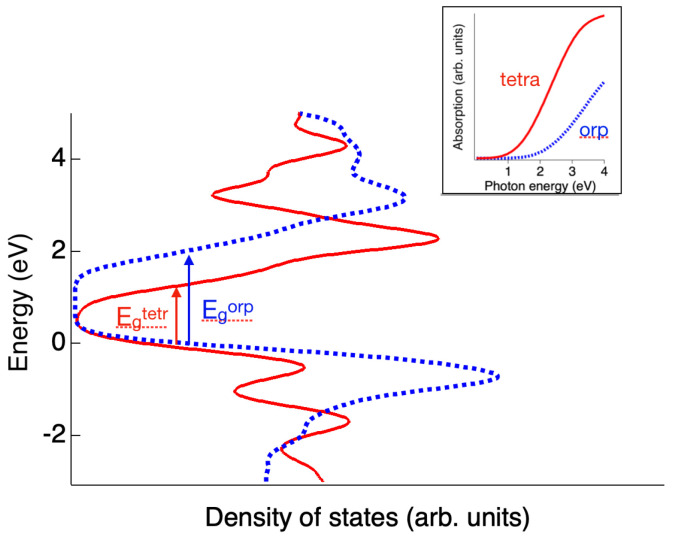
Calculated densities of states around the Fermi level for the orpiment and tetradymite phases demonstrate a significantly smaller band gap for the tetradymite phase. The inset shows the simulated absorption edges for the two phases with that for the tetradymite phase shifted to smaller energies, which corresponds to darkening.

It is informative to note that the average coordination number in the phase containing fragments of QLs is increased compared to the orpiment phase, which is in line with the experimental observation for elemental amorphous selenium [5]. Figure 4 compares the calculated densities of states for the orpiment and tetradymite phases. One can clearly see that the tetradymite structure with QLs has a significantly smaller band gap (0.7 eV vs. 1.8 eV). Also shown are the positions of the corresponding absorption edges with that for the tetradymite phase shifted to smaller energies, which corresponds to darkening. A fraction of 5% of the tetradymite phase would cause a 0.05 eV edge shift, which is in perfect agreement with the experiment. At the same time, it should be noted that the use of the PBE functional usually results in an underestimation of the band gap; sometimes, the difference between the calculated and experimental values can reach a value of 50%. Consequently, a quantitative comparison of the obtained results with the experiment is not very accurate.

**Figure 5:**
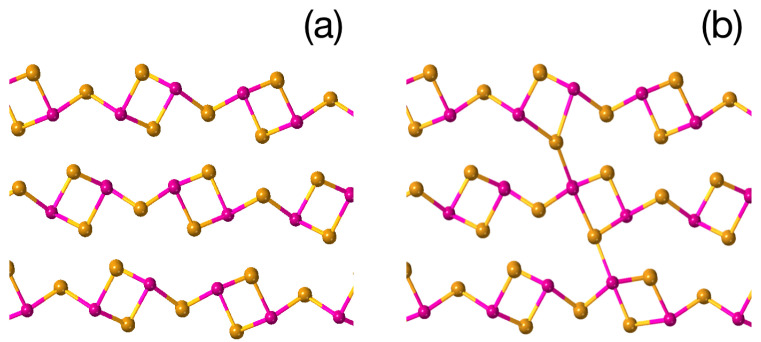
Schematics of the photo-induced structural change in the ${\mathrm{As}}_{2}S_{3}$ glass. Arsenic atoms are shown in violet and S atoms are yellow. (**a**)—ground state, (**b**)—photodarkened state.

It is interesting to note that a change between the two structures (locally) requires only minor atomic diffusion as illustrated in Figure 5.

The significantly lower optical gap for the structure with resonantly bonded complexes would account for the experimentally observed photodarkening. Furthermore, the slightly longer As-S interatomic distances in the extended QL structure as opposed to the classic covalent bond length (2.66 Å as opposed to 2.28 Å for the relaxed structures in our simulations) naturally accounts for the experimentally observed volume expansion. Since electrons located on *p*-orbitals have very anisotropic wave functions, the formation of such aligned complexes due to the directionality of the excitation source as well as their optical response to the probe beam are strongly anisotropic, which explains photo-induced anisotropy in chalcogenide glasses [55].

Here, we would like to note that the idea of the formation of tetradymite-like structural fragments with shorter and longer bonds was initially proposed by Dembovsky and Chechetkina as the self-organization of hypervalent bonds, or bond waves [56]. Recently, the important role of lone-pair electrons in the formation of multicenter bonds has been stressed in various publications [57,58], underscoring their significance for chalcogenide-based materials.

It is interesting to note some similarities between photodarkening in S- and Se-based chalcogenide glasses and the change in optical properties during the phase-change in tellurides [59] (e.g., for the case of GeTe-${\mathrm{Sb}}_{2}{\mathrm{Te}}_{3}$, where the band gap decreases from 0.7 eV to 0.5 eV upon crystallization). It was shown by different groups that the origin of the observed gap decrease was the formation of resonant bonds [60,61] in the octahedral local geometry of Ge atoms in the rock-salt-like crystalline phase as opposed to tetrahedrally coordinated Ge atoms satisfying the 8–N rule in the amorphous phase. In other words, the correlation between the formation of the extended multicenter, or hypervalent, or resonant, bonds and the decrease in the optical gap seems to be a general feature for various classes of chalcogenides.

Finally, we note that the model proposed in this work does not negate previously suggested models such as the photo-induced creation of valence alternation pairs. It is an independent and complementary mechanism that could account for the protodarkening process in chalcogenide glasses.

## 4. Conclusions

In conclusion, we propose that due to the presence of lone-pair electrons on chalcogen *p*-orbitals, aligned multicenter hypervalent complexes with resonant bonds can be formed in S- and Se-based chalcogenide glasses in the electronically excited state that are similar in their bonding nature to the atomic arrangement in quintuple layers of heavier chalcogenides. The lower optical gap for the resonantly bonded structure accounts for the reversible photodarkening and the increased interatomic distances provide a natural explanation for volume expansion, including direct surface modification by femtosecond laser, while the strong anisotropy of the multicenter fragments can account for photo-induced anisotropy. Quantitative estimates are difficult to make because the PBE functional used usually underestimates the gap value. The deeper understanding of the atomistic mechanism of the photo-induced changes in chalcogenide glasses proposed in this work will lead to their wider uses in infrared photonic devices, including polarization-sensitive ones.

## Data Availability

The data that support the findings of this study are available from the corresponding author upon reasonable request.
